# Equity in the utilization of physician and inpatient hospital services: evidence from Korean health panel survey

**DOI:** 10.1186/s12939-016-0452-3

**Published:** 2016-09-29

**Authors:** Ju Moon Park

**Affiliations:** Department of Urban Policy and Administration, Incheon National University, 119 Academy-ro, Yeonsu-gu, Incheon, 402-750 South Korea

**Keywords:** Physician visits, Inpatient hospital services, Health care system, Equity in utilization, Koreans

## Abstract

**Background:**

Little is known regarding equity in health care utilization among Koreans since 2008. This study examines the extent to which equity in the use of health care services has been achieved in Korea.

**Methods:**

Descriptive and logistic regression analysis was performed. The sample for this study was 17,035 individuals who participated in interviews.

**Results:**

Differences in need substantially account for the original differences observed between subgroups of Koreans. Need factors were important determinants of Koreans using physician and inpatient hospital services. Having income did not ameliorate the subgroup differences in the use of physician services. Nonetheless, having income remains an important predictor of physician utilization.

**Conclusions:**

The Korean health care system does not yield a fully equitable distribution of physician and inpatient hospital services. Health care reforms in Korea should continue to concentrate on insuring effective universal health care, implying that all population groups with need receive effective coverage.

## Background

The Republic of Korea has a National Health Insurance (NHI) system, covering almost the entire population. The National Health Insurance Corporation (NHIC), as the single payer, has responsibility for managing the NHI system. The Health Insurance Review and Assessment Service reviews the cost of health care benefits and evaluates the reasonableness of the health care services provided. Financing for the health care system is mainly funded through social health insurance contributions, government subsidies, and out-of-pocket payments by users of health services. In addition, the NHIC provides a range of useful information to beneficiaries regarding the availability of medical services, and, since 1 July 2008, has administered long-term care services for the elderly [1].

The introduction of a national health care insurance system in Korea in 1989 has improved Koreans’ access to medical care. According to the Korea Institute for Health and Social Affairs (1990, 2011) hospitalization increased from 48.5 to 95.0 per 1000 persons between 1989 and 2011. Physician visits increased from 40.4 to 90.1 per 100 persons between 1989 and 2011. The Korean National Health Insurance Corporation (2015) reported that 82.4 % of the respondents were satisfied with the services under universal health insurance.

However, the minimum role of the government in health care financing has resulted in relatively high out-of-pocket payments that may serve as a serious barrier to equal access to essential health care services [2]. The high out-of-pocket payment, including co-payments and uncovered services fees, has been believed to be one of the barriers to achieving horizontal equity in health care utilization in Korea [3]. Despite the universal health care system, the limited benefit coverage of the national health insurance program also threatens equal access to quality health care in Korea. Several issues should be addressed for further improvement of the Korean health insurance, including limited coverage, co-payments, and uncovered services fees [2–4]. Several studies have been conducted on equity in utilization of health services in Korea; however, most of them were conducted with the data from the 2006 Korean Longitudinal Study of Aging survey conducted before the long-term care insurance introduction [3, 5, 6] or with a sample of actual long-term care insurance beneficiaries [4]. Few studies have been conducted with representative national health survey data.

This study examines the extent to which equity in the use of two major sources of health care, physician and inpatient hospital services, has been achieved in Korea. The findings are based on the data from the 2011 Korea Health Panel Survey (KHPS). The Aday-Andersen behavioral model is used to guide empirical and normative assessment of equity under Korean universal health insurance system [7, 8]. Two principal questions with respect to equity of access in the use of physician and inpatient hospital services are addressed: (*a*) which subgroups of the Korean population are most likely to have utilized health care services, and (*b*) to what extent are the subgroup differences in utilization related to need? This study hypothesizes that the Korean health care system will be equitable.

## Methods

### Conceptual model

**Fig. 1:**
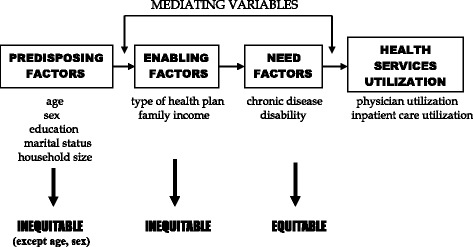
Conceptual framework for this study. The enabling and need factors are mediating variables that help to explain differences between subgroups that might be due to either equitable (*need*) or inequitable (*enabling*) factors. Age and sex serve as proxies for need because of the well-established relationships between illness patterns and age and sex. Other predisposing variables are, however, inequitable factors

The Aday and Andersen model [5, 7, 8] is used to guide the analyses (see Fig. 1). In this framework, a series of predisposing, enabling, and need factors are hypothesized to be predictive of utilization of services. The predisposing component includes those variables that describe the “propensity” of individuals to use services. The enabling component describes the “means” individuals have available to them for the use of services. The need component refers to the illness level, which is the most immediate cause of health care utilization [7]. Equity of access to care is measured based on the relative importance of need compared to other determinants of health care utilization. Access is equitable to the extent that predisposing, need-related demographic factors such as age and sex, as well as illness, account for health care utilization. Inequity is, however, suggested if services appear to be distributed on the basis of other predisposing, enabling variables, rather than need [8].

The analyses will focus on subgroup differences in whether an individual used health care services in the one year preceding the interview, and a systematic series of multivariate (logistic regression) analyses examining the extent to which these differences are explained by equitable (need-related) or inequitable (non-need-related) factors [5, 8].

### Study sample

This study used the data from the 2011 KHPS, which was released to the public in 2014. Baseline data from the 2011 KHPS were collected between May 12 and December 10, 2011, by the consortium of the National Health Insurance Service and the Korea Institute for Health and Social Affairs. Through a face-to-face interview survey, the KHPS provides information on demographic characteristics, service utilization behavior, medical expenditure, and health behaviors of the targeted households and their members. The sampling frame for the KHPS Household Component was drawn from respondents in accordance with the 2010 National Population and Housing Census. Sampling was done by stratified cluster sampling; the first step consisted of extracting sampling enumeration districts (clusters) based on the stratification variables (administrative divisions). The second step consisted of extracting sample households within the enumeration districts. The total number of samples is 17,035 individuals in 5741 households, and they participated in health interviews and health examination surveys.

The KHPS sampling weights incorporated adjustment for the complex sample design and reflected survey nonresponse and population totals from the current population survey; weights were applied in all statistical analyses to obtain nationally representative estimates.

### Measures

The dependent variable in this study was health services utilization. Two types of health care services were included: (*a*) physician services; and (*b*) inpatient hospital services. The measures of health services utilization were based on whether the sample person was reported to use physician and inpatient hospital services at least once in the past year.

Based on the literature review, predisposing factors are the indicators which are characteristically present prior to specific illness episodes [5, 8]. The literature supports the idea that several socio-demographic characteristics – sex, age, household size, education, and marital status – are associated with health care utilization as predisposing factors. I expected those who were older, women, educated, married, and living alone to be more likely to increase the utilization of physician and inpatient hospital services [5, 9, 10].

People with higher income tend to use more except those who spend down their assets to be eligible for Medicaid [5]. When interacted with insurance status, the effect of income moderated. The relationship between income and care is more complicated, for which further examination is necessary [11]. While total household income likely overstates the economic resources actually available to an individual, personal income likely understates the resources accessible to someone who is married. Thus the combination of spouses’ resources was deemed appropriate [12]. In this study, income refers to totals for individuals, if not married, or the couple, if married with spouse present in the same household. Economic theory suggests that in the presence of health insurance, the marginal cost of the insured good is lower, thus increasing quantity demanded of the good. Medicaid guarantees continued consumption of formal care, although on a somewhat more restrictive basis [11]. The enabling factors are major predictors explaining health care utilization [8]. As enabling factors affecting access to care, income level and type of health plan were included in this study. I expected people with income and insurance coverage to tend to use more physician services or inpatient hospital services [5, 8].

Need factors were the most directly associated with the accessibility to health services and reflect disease characteristics [13]. In this study, I included the presence of a chronic disease or disability. I expected people with disability or chronic conditions to be more likely to use physician and hospital services [5, 14].

There was no missing information on all predisposing, enabling, and need variables corresponding to the Aday/Andersen model. For the logistic regression analyses, the independent variables were re-coded to indicate dichotomies or a series of dummy variables (for variables with more than two categories, such as age group). The first category for a variable was coded 1 and the reference category for it (after “vs.”) was coded zero.

### Analysis plan and equity measurement

Descriptive statistics such as mean, standard deviation (SD), frequency, and percentage were used to analyze the individual characteristics of the sample. Logistic regression analysis was used to examine the relative importance of factors in predicting whether or not an individual used health care services.

To assess the relative importance of the respective predisposing, enabling, and need factors as predictors of utilization, the analyses were conducted in a series of stages. The predisposing variables were entered in analysis stage 1 to examine demographic subgroup differences. The need variables were entered in analysis stage 2 to examine the extent to which subgroup differences in stage 2 were reduced when variations in the need for care were controlled. At the final stage, the enabling factors were entered in analysis stage 3 to examine whether remaining subgroup differences were due primarily to the availability of personal or medical care resources. Based on the preceding analysis, I judge the extent to which the Korean health care system is equitable.

Equity in utilization of health care is often interpreted as persons in equal need of health care, who receive the equivalent treatment, irrespective of household income or socio-economic status. Therefore, according to the principle of horizontal equity, the measure for comparison among Koreans is inequality in using health care that remains after controlling for health care need differences [15, 16]. Nonetheless, need is mostly intractable in large-scale surveys and therefore, quantification remains a major challenge [17, 18]. In this study I utilized the measurement of equity which was verified empirically [5, 8]. The study measured the equity of access to medical care based on the relative importance of need compared to other factors of health services utilization; i.e. an equitable distribution of services would be reflected in demographic subgroup (except for age and sex) differences (stage 1) being largely explained by differences in need (stage 2). Empirically, this effect would be documented by the odds ratio becoming non-significant or remaining significant (*p* ≤ 0.05) but increasing (>) or decreasing (<) substantially (10+ %) in stage 2. An inequitable distribution of services would be reflected in the extent to which resource factors (such as health plan and income) have strongly independent or explanatory effects in accounting for variations in use (stage 3). Empirically, significant odds ratios for these factors in the stage 3 analyses would document their independent effects in predicting health care utilization. Substantial changes in the odds ratios for other variables (increasing or decreasing 10+ % or becoming non-significant) from stage 2 to stage 3 of the analyses, would attest to the explanatory effects of the enabling variables. That is, they help to account for (or explain) differences between demographic subgroups. In either case, the findings point to variations in use due to the availability of these resources.

The statistical significance of the odds ratios (the ratio of the likelihood that one age group, e.g., 55 + years, has access compared to another age group, e.g., 20–54 years) was examined to evaluate the impact of the predisposing, enabling, and need factors at each stage. Changes in the magnitude or significance of the odds ratios in the successive stages were used to identify those factors that might help to account for subgroup differences in the probability of using health care services.

## Results

The demographic and socioeconomic characteristics along with health care utilization are presented in Table 1. The average age of the respondents was 43.2 ± 20.2 years old. 51.3 % of the respondents were female; 53.0 % had a spouse; 5.3 % lived alone. 29.7 % of the respondents had 6 years of schooling or less; 41.1.0 % had 7–12 years of schooling; 29.2 % had 13 years of schooling or more. 94.7 % of the respondents had health insurance; 5.3 % had medical aid.

**Table 1:** Characteristics of respondents (*n* = 17,035)

Study variable	Value
Predisposing characteristics	
Age, years [mean (±SD)]	43.2 (±20.2)
Female, %	51.3
Education (graduation), %	
0–6years	29.7
7–12years	41.1
13 +	29.2
Having a spouse, %	53.0
Living alone, %	5.3
Enabling characteristics	
Type of health plan, %	
Health insurance	94.7
Medical aid	5.3
Income, 1,000 won^a^ [mean (±SD)]	2,255.5 (±1,904.5)
Health needs	
Having a chronic disease, %	46.8
Having a disability, %	5.8
Health care services	
Physician visit	80.9
Hospitalization	9.3

^a^Korean monetary unit ($US 1 = KRW 1,150)

The yearly average income of respondents or their family members spent was 22,550,000 ± 19,045,000 won. The respondents with at least one chronic disease accounted for 46.8 %, taking up a large portion. Approximately 94.2 % of the respondents responded that they had no disability; 5.8 % had a disability.

Table 1 also shows the distribution of services utilization by the respondents. Most of the respondents (80.9 %) were using physician services. By contrast, only 9.3 % were using inpatient hospital services.

### Physician utilization

The odds ratios for physician utilization, simultaneously adjusted for multiple independent variables, are presented in Table 2. After adjusting for an array of predisposing factors (stage 1), Koreans who were more likely to have used physician services included children (<19 years), older adults (>55 years), women, those who were living alone, those who had schooling of 7 years or more, and those who were unmarried or divorced or separated.

**Table 2:** Multivariate logistic regression analysis of predictors of physician utilization for Koreans, weighted (2011)

Determinants	Physician utilization					
	Stage I		Stage II		Stage III	
	Odds ratio	*p*	Odds ratio	*p*	Odds ratio	*p*
	(95 % CI)		(95 % CI)		(95 % CI)	
Predisposing:						
Age (years)						
20–54 vs 0–19	0.313	<0.0001	0.281	<0.0001	0.224	0.0014
	(0.269–0.365)		(0.240–0.330)		(0.089–0.561)	
20–54 vs 55 +	0.344	<0.0001	0.736	0.0002	0.517	<0.0001
	(0.299–0.397)		(0.626–0.865)		(0.409–0.653)	
Sex						
Female vs Male	2.403	<0.0001	2.275	<0.0001	3.053	<0.0001
	(2.178–2.651)		(2.054–2.519)		(2.606–3.578)	
Education						
7 + vs 0–6 years	3.808	<0.0001	3.416	<0.0001	1.783	0.0019
	(3.192–4.544)		(2.843–4.104)		(1.238–2.568)	
Marital status						
Others vs Married	3.210	<0.0001	2.764	<0.0001	2.664	<0.0001
	(2.881–3.577)		(2.465–3.099)		(2.289–3.101)	
Household size						
Living alone vs Others	2.809	<0.0001	2.794	<0.0001	1.805	0.0005
	(2.353–3.353)		(2.314–3.373)		(1.292–2.521)	
Need:						
Chronic disease						
Yes vs No			5.010	<0.0001	5.587	<0.0001
			(4.402–5.701)		(4.717–6.618)	
Disability						
Yes vs No			1.192	0.2681	1.694	0.0289
			(0.902–1.573)		(1.056–2.718)	
Enabling:						
Income						
0–30 million vs 30 million +					1.207	0.0215
					(1.028–1.417)	
Type of health plan						
Health insurance vs Medical aid					0.699	0.1706
					(0.419–1.167)	

These relationships were re‐examined, adjusting for need (stage 2). Koreans who had chronic disease were five times more likely to have used physician services than their counterparts. Chronic disease had a notable impact on the odds ratios of physician utilization for the predisposing variables entered in stage 2; all the demographic subgroup differences (except for those 0–19 versus adults 20–54) were in general narrowed in stage 2 when adjusted for need. Specifically, all the predisposing variables selected in this study became significant at the 0.05 level. The findings suggest that chronic disease remains an important predictor of the use of physician services among Koreans (stage 2).

The impact of the enabling factors was examined in stage 3. The enabling factors were income lower than 30 million won vs. >30 million won and health insurance vs. Medical aid. Those who had less than 30 million won of total annual income were much more likely to have used physician services than those who had income higher than 30 million won. Adjusting for having income lower than 30 million won vs. >30 million won had had an impact on the odds ratios of physician utilization for the predisposing and need factors. The remaining subgroup differences were not ameliorated once the resource variables were taken into account. The odds ratios for other variables (except for education, marital status, and household size) were rather increased from stage 2 to stage 3 of the analyses. After the new set of enabling variables was added, disability became significant. Disability as well as chronic disease was important predictors of the use of physician services among Koreans once the resource variables were taken into account.

In summary, having income lower than 30 million won vs. >30 million won did not fully ameliorate the remaining subgroup differences in the use of physician services among Koreans, observed in stage 3. Nonetheless, having income lower than 30 million won vs. >30 million won remains a significant independent determinant of physician utilization.

### Inpatient care utilization

Table 3 shows that after simultaneously adjusting for the predisposing factors (stage 1), those who were more likely to have used inpatient hospital services included those aged 55+, women, those who had schooling of 7 years or more, and those who were unmarried or divorced or separated.

**Table 3:** Multivariate logistic regression analysis of predictors of inpatient care utilization for Koreans, weighted (2011)

Determinants	Inpatient care utilization					
	Stage I		Stage II		Stage III	
	Odds ratio	*p*	Odds ratio	*p*	Odds ratio	*p*
	(95 % CI)		(95 % CI)		(95 % CI)	
Predisposing:						
Age (years)						
20–54 vs 0–19	1.121	0.2910	0.979	0.8478	0.713	0.5902
	(0.907–1.387)		(0.791–1.212)		(0.208–2.441)	
20–54 vs 55 +	0.493	<0.0001	0.664	<0.0001	0.723	0.0032
	(0.429–0.566)		(0.575–0.766)		(0.583–0.897)	
Sex						
Female vs Male	1.260	<0.0001	1.237	0.0003	1.272	0.0149
	(1.126–1.411)		(1.103–1.399)		(1.048–1.544)	
Education						
7 + vs 0–6 years	1.55	<0.0001	1.405	<0.0001	1.256	0.0567
	(1.360–1.780)		(1.230–1.605)		(0.089–0.561)	
Marital status						
Others vs Married	1.556	<0.0001	1.480	<0.0001	1.324	0.0125
	(1.353–1.825)		(1.270–1.726)		(1.062–1.651)	
Household size						
Living alone vs Others	0.887	0.4007	0.814	0.1482	1.231	0.4641
	(0.672–1.172)		(0.616–1.076)-		(0.706–2.146)	
Need:						
Chronic disease						
Yes vs No			1.997	<0.0001	2.205	<0.0001
			(1.735–2.298)		(1.782–2.729)	
Disability						
Yes vs No			1.880	<0.0001	1.910	0.0002
			(1.564–2.259)		(1.353–2.695)	
Enabling:						
Income						
0–30 million vs 30 million +					0.904	0.3931
					(0.717–1.140)	
Type of health plan						
Health insurance vs Medical aid					0.840	0.4393
					(0.540–1.307)	

Koreans who had chronic disease were much more likely to have used inpatient hospital services than their counterparts (stage 2). Those who had a disability were much more likely to have used inpatient hospital services than Koreans who had no disability. The need variables related to disability and chronic disease had a notable impact on the odds ratios of hospitalization for the predisposing variables entered in stage 2.

All the need variables had a significant impact on the subgroup differences in hospitalization (see Table 3). All the demographic subgroup differences (except for those who were living alone versus others) were reduced in stage 2; that is, the odds ratios shifted toward unity. The findings suggest that need factors such as chronic disease and disability remain important predictors of the use of inpatient hospital services among Koreans.

The impact of the enabling factors was examined in stage 3. Adjusting for having resource availability had no impact on the odds ratios of inpatient hospital services utilization for the predisposing and need factors. The findings suggest that resource availability related to type of insurance coverage and income level do not remain important predictors of the use of inpatient hospital services (stage 3). Changes in the odds ratios from stage 2 to stage 3 of the analyses were extended for female versus male and living alone versus others, but other demographic subgroup (those over 55 versus adults 20–54, those who were married versus others, and those with more than seven years of schooling versus those with fewer than seven years of schooling) differences were reduced when adjusted for need (stage 3). The need variables like chronic disease and disability remain important predictors of the use of inpatient hospital services among Koreans.

In summary, having personal or health care resources such as income and insurance did not fully ameliorate the remaining subgroup differences in the utilization of inpatient hospital services among Koreans, observed in stage 3. They were not significant independent determinants of inpatient hospital service utilization.

## Discussion

Korea achieved its universal health insurance coverage including long-term care. Access to health care has been improved, but there still remain populations at risk in Korea. The research reported here addresses this issue.

The results of this study do not fully support the hypothesis that the Korean health care system will be equitable. In the multivariate analysis, this study reveals that need factors such as disability and chronic disease were important determinants of Koreans using inpatient hospital services and physician services. Differences in need substantially account for the original differences observed between subgroups of Koreans (see Tables 2 and 3). The results also establish that having income lower than 30 million won vs. >30 million won did not fully ameliorate the remaining subgroup differences in the use of physician services among Koreans. Nonetheless, having income lower than 30 million won vs. >30 million won remains an important independent predictor of physician utilization.

This study indicated that in Korea, children (<19 years), older adults (>55 years), women, and well-educated persons were more likely than their counterparts to have used physician and inpatient hospital services. Physician utilization is significantly related with age; U-shape curve. Single persons were more likely than their counterparts to have used physician and inpatient hospital services. Unlike previous studies [5, 9, 10], those who were married are not more likely than their counterparts to increase the utilization of physician and inpatient hospital services. This may be explained by the fact that married Koreans in traditional patriarchal families, especially women, may have been socialized to place the needs of other family members before their own, which may have hindered them from seeking medical services [14]. In this study, the variables indicating a child under 20 years of age versus adults 20–54, and household size are generally insignificant for hospital utilization, but significant for physician utilization. However, the variables indicating older adults aged 55+ versus adults 20–54, gender, and marital status were significant for the utilization of physician and inpatient hospital services.

The Korean universal health insurance program does not yield a fully equitable distribution of services for Koreans, who were reported in the existing health care literature as a group with higher needs, but limited access to care [3, 5, 6, 14]. This study indicated that the use of primary care was found inequitable in favor of the less well-off. This finding may imply that lower income groups were over-utilizing the services of a general practitioner. Similar concerns about higher service utilization have been born out in the medical aid program, which subsidizes health insurance co-payments for the low-income population in Korea [19]. Moreover, such higher use of service by the subsidized is possible as the Korean health care system has no gate-keeping or care management system. The government recently introduced a care management program to monitor and guide medical aid beneficiaries with a high utilization of health care [4], but its effectiveness is still under evaluation.

This study contributes to the existing literature on health care equity: to my knowledge, this study is the first study to examine the extent to which equity in the use of health care services has been achieved in Korea, using data from the national healthcare survey conducted after the introduction of the long-term care insurance. There is literature on health care equity more generally. For example, Thailand, the first non-OECD country to embrace Universal Health Coverage (UHC), has shown remarkable improvements with regards to inequality in outpatient care use, though the use of hospital care became more concentrated among the better off between 2001 and 2005 [20]. In China, inequities in utilization of outpatient and inpatient care have also declined significantly in the recent years due to the increased insurance coverage and primary health care [21]. In Italy, use of primary care was found inequitable in favor of the less well-off, and hospitalization was essentially equitable [22]. Owing to universal health care in Korea, the quantity of health care utilization is equitable compared to other developed countries, showing neutral or pro-poor inequalities for both primary and secondary care utilization [23]. Few studies, however, have examined the extent to which equity in the use of two major sources of health care, physician and inpatient hospital services, has been achieved in Korea.

Our findings are consistent with evidence that shows reduced inequities in health care use by increasing access to health care in several countries [3]. Methodologically, I used large-scale survey data collected by the Korea Institute for Health and Social Affairs and the National Health Insurance Corporation in 2011. I also examined various predisposing, enabling, and need variables associated with health services utilization based on a literature review, and also evaluated the equity of the Korean health care system based on the relative importance of need compared to other factors of health services utilization. There are also several limitations. The analysis model, as used in this study, was limited to the data collected by the Korea Health Panel Study in 2011. There is difficulty that arises as the result of using the model with the secondary data, e.g., as for the study design, none of the established association can be inferred as a cause-effect relation. Also, the data did not include place of residence that might reveal plausible geographical differences in equity. In previous studies by Park [5], place of residence was found to influence health care utilization. There could also be unobserved factors associated with health care utilization in this study due to the limitation of the data. These variables should be included in future study.

## Conclusions

This study provides evidence that the Korean universal national health care system does not yield a fully equitable distribution of physician and inpatient hospital services. In order to address the persistence of inequities in utilization of health care, it would be necessary to insure effective universal health care, implying that all population groups with need receive effective coverage.

Variations in the patterns of use of physician and inpatient hospital services for certain subgroups of Koreans, i.e., men, poorly educated persons, and/or married people, point to the fact that non-financial policy options or modifications of the existing financing system may be required to enhance access for these groups.

In summary, this study has suggested that health care reforms in Korea should continue to concentrate on insuring effective universal health care, implying that all population groups with need receive effective coverage. Further research is also needed to understand those who have lower income, chronic diseases, and disability, why the access barriers may exist for selected demographic subgroups, i.e., those who were men, less-educated, and married.
